# A Robust Deep Learning Ensemble-Driven Model for Defect and Non-Defect Recognition and Classification Using a Weighted Averaging Sequence-Based Meta-Learning Ensembler

**DOI:** 10.3390/s22249971

**Published:** 2022-12-17

**Authors:** Okeke Stephen, Samaneh Madanian, Minh Nguyen

**Affiliations:** Computer Science & Software Engineering, Auckland University of Technology, Auckland 1010, New Zealand

**Keywords:** deep learning ensemble, defect recognition and classification, visual inspection, industrial products, product quality control, conv-LSTM

## Abstract

The need to overcome the challenges of visual inspections conducted by domain experts drives the recent surge in visual inspection research. Typical manual industrial data analysis and inspection for defects conducted by trained personnel are expensive, time-consuming, and characterized by mistakes. Thus, an efficient intelligent-driven model is needed to eliminate or minimize the challenges of defect identification and elimination in processes to the barest minimum. This paper presents a robust method for recognizing and classifying defects in industrial products using a deep-learning architectural ensemble approach integrated with a weighted sequence meta-learning unification framework. In the proposed method, a unique base model is constructed and fused together with other co-learning pretrained models using a sequence-driven meta-learning ensembler that aggregates the best features learned from the various contributing models for better and superior performance. During experimentation in the study, different publicly available industrial product datasets consisting of the defect and non-defect samples were used to train, validate, and test the introduced model, with remarkable results obtained that demonstrate the viability of the proposed method in tackling the challenges of the manual visual inspection approach.

## 1. Introduction

Sustaining quality standards is a crucial task for every industry, and visual inspections deal with the detection of defects from manufactured products for quality control. Quality inspections can be conducted at any stage of the industrial production circle, such as product components, products within the manufacturing lines, incoming material, or finished products. Inspection examines products to determine those that meet the set standards and those that deviate from the set quality requirements, paving the way for the rejection of faulty products and progression to the next stage of those that conform to the set standards [1]. In situ or in-process inspections are standard practices conducted during industrial parts and other product manufacturing processes [2]. Manual inspections for defects are characterized by challenges such as boredom of inspection operators, failure to meet production targets, bias, inadequate inspection skillset, subjective judgements, etc.

**Figure 1:**
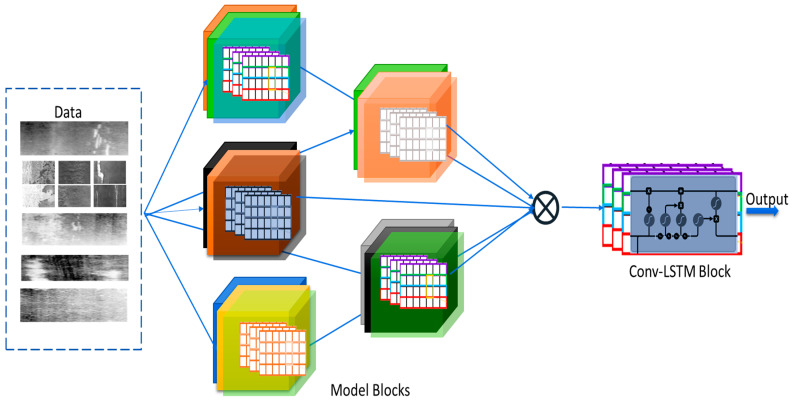
The flow sketch of the proposed Weighted Averaging Sequence-based Meta-learning Ensembler.

The limitations of the human-oriented industrial visual inspection for faulty product identification could be addressed through independent, intelligent models and computer vision algorithms. In recent years, intelligent machine vision models have become desirable in tackling high costs and other shortcomings of human-driven defect recognition and analysis processes. Deep learning (DL) models, in particular, convolutional neural networks (CNNs), have been increasingly used in the automation of inspection processes [3,4]. CNN-driven models have proven effective in performing visual inspections by recognizing, classifying, and detecting defects and non-defects in objects of interest [5]. Despite the remarkable performance of deep learning techniques in recent times, significant issues and challenges, such as model robustness, performance accuracy, and efficiency, are still abound. Therefore, in this work, we propose a robust deep-learning method driven by the model ensemble concept with a sequence-enabled meta-learning unifier to perform the recognition and classification of an industrial product for defect identification (see Figure 1).

This article section presents an overview of the current state and the limitations of human-in-the-loop industrial product inspection systems. In Section 2, relevant and related literature were explored, ranging from the use of complex multilayer CNN architecture, conditional random field (CRFs) algorithm with CNN, fully convolutional network (FCN), meta-learning CNN architectural framework, deep convolutional sparse-coding-based network, etc. Section 3 provides the theoretical background and the method adopted in the study, while the experimental procedure used in the study is elaborated in Section 4. In Section 5, the results obtained from the study are presented and concisely explained. Comparisons with related works are also made in Section 5, while the study’s conclusion is presented in Section 6.

## 2. Related Works

Recently, product defect identification and classification for visual inspections have attracted considerable research interest. He et al. [6] and Borji et al. [7] deployed a deep-learning model based on the LeNet network structure [8]. Their proposed framework detects defects in industrial products using a complex multilayer CNN architecture to extract defect image features and then a full end-to-end training process to learn and classify the defects. In another work on defect spotting and the classification of products, a CNN model and a conditional random field (CRFs) algorithm were combined to train and optimize a built DL network prediction process [9]. Xue and Li (2018) deployed a region-based fully convolutional network (FCN) DL model to build an intelligent classification and detection model for rapid tunnel lining defect detections. 

Furthermore, Bartler et al. [10] proposed a DL-based classification pipeline to identify solar cell defects automatically. A meta-learning CNN architectural framework was introduced to perform a multi-target concrete defect classification in concrete bridge image frames [11,12]. In another work, a DL model was deployed to ensure sustainable transportation by developing a model that fused the features of two models for high accuracy in classifying defects on rail tracks [13]. Krummenacher et al. [14] proposed a wheel defect identification system based on machine learning methods on railway wagons for easy damage recognition on rolling stocks and railway infrastructures. A deep convolutional sparse coding-based network was deployed to perform tire defect classification tasks to ensure an efficient quality control process [15]. A weld defects classification framework driven by transfer learning and activation features of deep learning was proposed to detect defects on industrial weld X-ray images for a rapid, nondestructive test process [16].

Konovalenko et al. [17], in their work on defect classification, proposed a deep residual neural network-based model to classify defects and non-defects on steel surfaces. In a similar study, a time-efficient steel surface defect classification built with a completed local binary pattern was introduced by Luo et al. [18]. Wang et al. (2021) presented a graph convolution network-based semi-supervised model to learn the inter-class similarities and intra-class variations in surfaces for fault and non-fault recognition and classifications. With the aid of a hybrid chromosome genetic algorithm, Hu et al. [19] developed a large-scale strip steel surface defect classification framework. Additionally, an automatic PCB defect classification, analysis, and inspection system was introduced by Deng et al. [20], and Zhang et al. [21] proposed a multi-label class classification of PCB defects using a multi-task convolutional neural network framework. For a micro-defect diagnosis on piston throats, Chen et al. [22] proposed a SMOTE in conjunction with a new model selection method utilized on the active learning of the SVM algorithm (E-SVM-AL). Additionally, an image processing-based piston surface defect recognition system combined different strategies, such as edge detection, threshold segmentation, and morphological operations, to recognize defects on piston surfaces [23]. Furthermore, Nikolić et al. [24] introduced a deep learning-based classification methodology to detect the porosity defects in aluminum alloys, and Habibpour et al. [25] proposed an uncertainty-aware deep learning model to detect defects in industrial casting products. Despite these studies on defect recognition and classification, little effort has been made on the robust model for the defect spotting in products; therefore, we propose a weighted sequence-based meta-learning ensemble on a collection of models aggregated together to learn the class and interclass similarities and dissimilarities in objects for defect and non-defect separation.

## 3. Theoretical Background and Method

This section presents the underground theoretical method for the proposed defects recognition and classification framework. Let $D=\left\{(d_{z},\;c_{z})\;1\leq z\leq N\right\}$ represent the dataset consisting of N number of training samples, with $c_{z}=\left\{1,\;2,\ldots ,C\right\}$ their corresponding class labels and C the total sum of the classes. Then, the proposed model contains M different numbers of deep learning models fused with convolutional LSTM layers that learn from the meta-features emanating from the various participating models for superior performance. The proposed method can ensemble different numbers of given CNN models. However, in this study, we used M=5 number of models for the metal surface defects classification and M=6 for the other datasets. In the deep learning ensemble process, the resultant features R from the various model is expressed as $R=\left[r_{1}r_{2},\ldots ,r_{n}\right]$. During training, a forward propagation process is conducted in each epoch to generate features from each co-learning model and then fussed together by the integrated sequence-based convolutional LSTM layers. 

### 3.1. The Contributing CNN Models

In this investigation, we crafted a unique base model and adopted four other state-of-the-art convolutional neural network-based models: Inceptionv3 [26], DenseNet [27], Xception [28], and MobileNet [29]. The built base model contains four significant layers and sublayers, as shown in Table 1, with 223,873 total parameters used in the model training process. The respective feature extractors in the CNN architecture have conv2 × 3, 32; conv2 × 3, 64; and conv2 × 3, 128 layer sizes, as well as a 2 × 2 max pooling layer size between the 1st and the 2nd layers. A Relu activator was equally used in the 1st three layers (see Table 1), and a stride of 2 was used across all the layers excluding the last layer.

Inception-v3, which belongs to the inception model group, consists of a label smoothing mechanism, factorized 7 × 7 convolutions, and an auxiliary classifier that channels training data label details from the top to the lower ebb of the network [26]. The DenseNet (densely connected convolutional networks) [27], on the other hand, is a variant of the deep CNN model that consists of dense blocks and uses dense connections between layers in the network to propagate information across the network. Furthermore, the Xception model depends on depth-wise separable convolution layers to compute the spatial information from the training and validation data. Finally, on the co-learning models, the MobileNet was initially designed and built for mobile applications [29]; however, many applications have adopted the framework to solve different scientific problems [30,31,32].

All the adopted models in this study were ImageNet dataset pre-trained, but the decision layers were chopped off during our experiments because the models were pre-trained initially to classify objects of 1000 classes. During the training process in the experiments conducted in this study, the meta-features that emanated from the various models were concatenated via the integrated weighted averaging sequence-based meta-learning ensemble, which then performed the final classification tasks. The convolution components of the models were useful in extracting the features from the defect and non-defect data samples. The features fed to the unification framework are called meta-features and are significantly valuable for distinguishing the defective and non-defectives data samples. The lower layer of the networks extracted the local image data features, while the higher layers extracted more semantic meta-features through convolution operations.

### 3.2. The Weighted Averaging Sequence-Based Meta-Feature Learning Derivative

In the weighted averaging ensemble strategy, the final model’s classification report was acquired by obtaining the outputs of the various contributing models and averaging the results with some weight inducements for better predictive performance. This, in particular, motivated us to adopt this approach because of its robustness and ability to handle imbalanced datasets, such as the ones used in this investigation. The weighted averaging sequence-based meta-feature learning component of the proposed method was inspired by the work of Shi et al. [33], in which the meta-features from all the inputs $I_{1},\cdots I_{t,\;}$ the output of the cells $O_{1},\cdots O_{t},$ the hidden states $H_{1},\cdots H_{t}$, and the gates $g_{t},l_{t},m_{t}$ of the ConvLSTM layer were 3D tensors that enable our proposed method to learn the spatial meta-features of the defect and non-defect samples in the final aggregate layers (see Equations (1)–(5)). In other words, the ensemble layer of the proposed method consists of ConvLSTM layers that possess convolution operators which join the features emanating from the various co-learning models. The ConvLSTM layer component of the proposed weighted averaging sequence-based meta-feature learning is expressed as:

(1) $$g_{t}=\sigma \left(W_{ig}\cdot I_{t}+W_{hg}\cdot \;H_{t-1}+W_{og}\cdot O_{t-1}+k_{g}\right)$$

(2) $$l_{t}=\sigma \left(W_{il}\cdot \;I_{t}+W_{hl}\cdot \;H_{t-1}+W_{ol}\cdot O_{t-1}+k_{l}\right)$$

(3) $$O_{t}=l_{t}\cdot O_{t-1}+g_{t}\cdot tanh\left(W_{io}\cdot \;I_{t}+W_{ho}\cdot \;H_{t-1}+k_{o}\right)$$

(4) $$m_{t}=\sigma \left(W_{im}\cdot \;I_{t}+W_{hm}\cdot \;H_{t-1}+W_{om}\cdot O_{t}+k_{m}\right)$$

(5) $$H_{t}=m_{t}\;\cdot tanh\left(O_{t}\right)$$

In training the model, padding is required before the application of convolution operations to guarantee that the same number of matrix computations are conducted as the inputs possessed by the state. In the ConvLSTM layer, all the states in the LSTM are initialized to zero before the arrival of the first input, and a zero-padding was used in the hidden states in this study, so that the boundary points of the training dataset were computed differently for prompt learning of the intra-class differences in the defect and non-defect samples.

## 4. Experiments

### 4.1. Dataset Preparation Process

We adopted four datasets consisting of different types of defect and non-defect samples to train and validate the proposed method for robustness and performance. The first dataset employed in this study is the NEU (Northeastern University) surface defect dataset consisting of six distinct classes of typical surface defects [34]. The data were collected and made available for research from hot-rolled steel strips with patches (Pa), pitted surface (PS), rolled-in scale (RS), crazing (Cr), scratches (Sc), and inclusion (In) as defect classes. The dataset initially contained 1,800 grayscale images of 200 × 200 size. During the experiments in this study, the dataset was split into 1152 samples for the train set, 288 samples for validation, and 360 for testing the final trained model. The second dataset [35] used in the experiment consisted of 512 × 512 grey scale-size images of submersible pump impellers with defects and non-defect samples available publicly for research. The total number of images was 7348 samples, which were reduced to 300 × 300 grayscale sizes and split into 4644 training samples, 1989 validation samples, and 715 samples for testing the proposed model.

Furthermore, the third dataset employed in this investigation was the printed circuit board (PCB) industrial dataset [36], which comprised 1500 images of defect and non-defect samples. The data were obtained from linear scan CCD processes in the resolution range of 48 pixels per 1 mm. The dataset was cross-examined and certified for suitability for training and validating the proposed model and was split into 892 training samples, 223 validation samples, and 180 test samples. Finally, the fourth dataset used in the experiments was the piston image dataset from industrial mechanic components with shaped-out, greasy, broken, fallen, rust stains, and oily class samples [37]. The dataset contained 285 samples and was collected during the AC’s pistons production process. During the experiments in this study, the dataset was divided into 173 samples for training, 42 validation samples, and 70 test samples.

### 4.2. Experimental and Evaluation Metrics

We implemented the proposed DL method using a high-end computing resource integrated with two GPU cores, each having a 12GB video card and a RAM size of 32GB. Keras and TensorFlow open-source DL libraries, in conjunction with other supportive python modules, such as NumPy, pandas, matplotlib, sklearn, etc., as well as a Linux operating system, were also used for the implementation of the introduced DL method. There were 30 epochs, each involved in the training and validating of the individual models used in the experiments. Furthermore, a binary cross-entropy loss function was used for the datasets, except for the NEU categorical dataset; thus, a categorical cross-entropy loss function was used. A 1 × 10 − 3 × 0.9 learning rate scheduler with a 1 × 10^−4^ learning rate Adam optimizer was employed to train and validate the models. To help boost the performance of the models’ entire training and learning process, standard augmentation techniques were deployed to artificially increase the training data (i.e., horizontal flipping, random cropping, rotation, and shear range). At the end of the training and validation process of the selected models, the meta-features obtained from the models were aggregated using the weighted averaging sequence-based meta-feature learning ensembler to form the new proposed model.

Different DL model evaluation metrics were employed to thoroughly examine the results obtained from the experiments run in this investigation. One such metric is the Cohen kappa score, which calculates the inter-rater trustworthiness of the proposed model. Additionally, Matthew’s correlation coefficient (MCC) estimates the quality of association between the pairs of the defect and non-defect samples. The mean square error measures the mean square of the difference between the actual data samples and the predicted samples. In contrast, the mean square log error obtains the relative error between the actual defective and non-defect data samples and the predicted samples. We further extracted the precision, recall, F1-score, and weighted average scores of the individual models and the proposed model, thus solidifying the results of the proposed method.

## 5. Results

We first constructed a unique, but efficient, custom CNN model to handle the problems of dataset limitations effectively. The CNN model is simple and unique because it consists of a few layers and parameters (see Table 1), but proactively learns the defect’s features and non-defect data samples. Additionally, we adopted the transfer learning (TL) concept on the other pre-trained models selected in this study. We first trained the custom model, Inceptionv3, Xception, DenseNet, and MobileNet, with the data samples allotted for training. Then, we fused the outputs of the models using the introduced weighted averaging sequence-based meta-feature learning ensembler to form an entirely new model for superior performance. A comparative performance of each of the adopted co-learning models with the proposed model was conducted and tabulated accordingly (see Table 2, Table 3, Table 4 and Table 5). The classification performance, with respect to the Cohen kappa (Kp), Matthew’s correlation coefficient (MCC), accuracy, mean square error (MSE), and mean square log error (MSLE), using the NEU dataset, is shown in Table 2 below.

**Figure 2:**
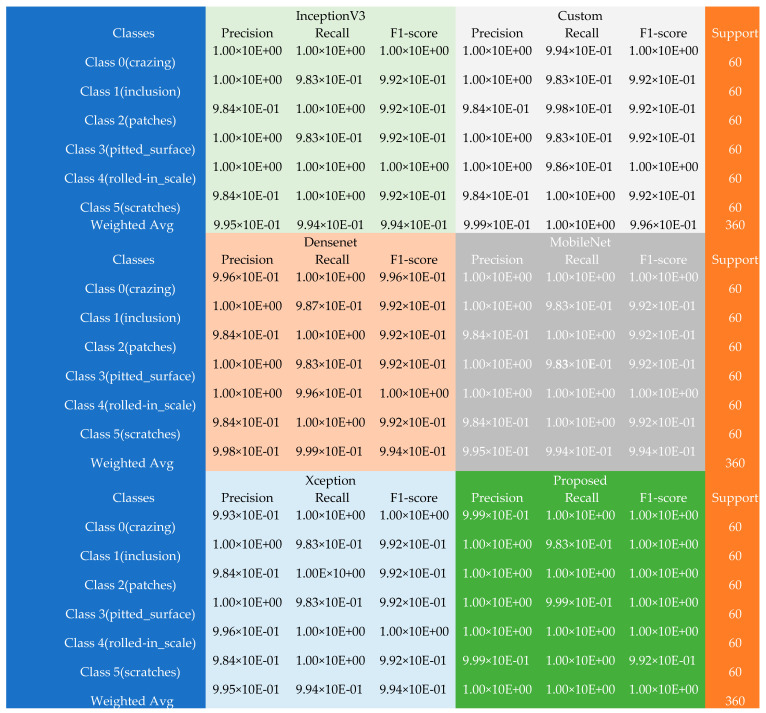
The confusion matrix table using the NEU dataset.

As tabulated in Table 2, the classification performance significantly improved after the models’ ensemble process with approximately 9.99 × 10^−1^ KP score, 1.00 × 10^+00^ MCC, 1.00 × 10^+00^ accuracy, and low MSE and MSLE of 3.47 × 10^−4^ and 3.48 × 10^−6^, respectively. More vividly, the performance accuracy scores rose from 9.94 × 10^−1^ (Inceptionv3), 9.94 × 10^−1^ (both the Custom and DenseNet), and 9.93 × 10^−1^ (MobileNet) to 1.00 × 10^+00^ for the proposed method. Detailed performance of the proposed method is shown using the confusion matrix table having the experiments’ precision, recall, and F1-scores (see Figure 2).

The rows in the confusion matrix table correspond to the various classes of the NEU dataset, i.e., class 0 equals the crazing data samples, class 1 represents the inclusion samples, and class 2 represents the patched samples. The pitted_surface samples are denoted by class 3, while the rolled-in_scale samples are defined by class 4 and class 5, represented by the scratch samples. Additionally, the overall weighted average is found in the rows. According to the confusion matrix table, the InceptionV3 model returned close to 100% scores in precision, recall, and F1-score across the samples, with a weighted average precision of 9.95 × 10^−1^, weighted average recall of 9.94 × 10^−1^, and a weighted average F1-score of 9.94 × 10^−1^. The scores were similar across the other participating models, but the proposed model yielded a superior performance, with a weighted average precision, recall, and F1 score of approximately 100%. Furthermore, the results obtained using the piston dataset are shown in Table 3.

From Table 3, both the DenseNet and MobileNet recorded the least Kp, MCC, and accuracy scores. The Inceptionv3 followed this with 7.90 × 10^−1^ Kp, 7.90 × 10^−1^ MCC, and 9.14 × 10^−1^ accuracy scores, while the crafted custom model returned accuracy scores of 9.43 × 10^−1^, 8.51 × 10^−1^ Kp, and 8.61 × 10^−1^ MCC. In continuation, the Xception model produced the second-best performance, with 9.66 × 10^−1^ Kp, 9.66 × 10^−1^ MCC, and 9.14 × 10^−1^ accuracy. Meanwhile, the proposed model produced the overall best performance, with 9.80 × 10^−1^ Kp, 9.72 × 10^−1^ MCC, and 9.49 × 10^−1^ accuracy, showing a remarkable performance accuracy gain over the various participating models.

**Figure 3:**
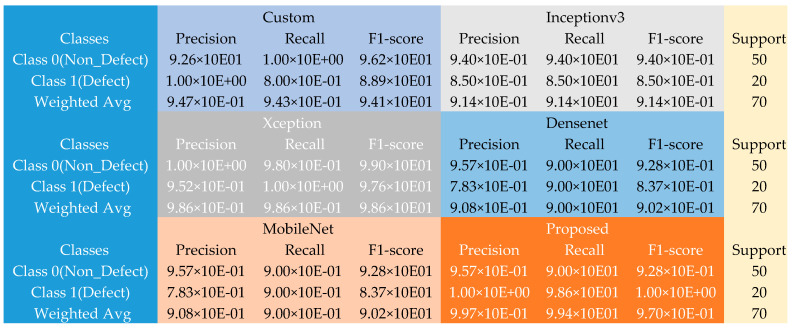
The confusion matrix table using the piston dataset.

Figure 3 shows more detailed results extracted from the various experiments conducted with the piston dataset. Given the 50 non-defect and 20 defect samples, the introduced method yielded a tremendous performance against the different pre-trained models and the typical model constructed during the experiments. The custom model produced weighted average precision, recall, and F1 scores of approximately 95% each across the 70 defect and non-defect test samples, and the Inceptionv3 model output weighted average precision, recall, and F1 scores of about 91% each; the Xception model generated weighted average precision, recall, and F1 scores of approximately 98.6% each. In contrast, the DenseNet and MobileNet produced weighted average precision, recall, and F1 scores of roughly 91% each across the 70 defect and non-defect test samples. In comparison, the proposed method outclassed these models by producing an improved weighted average precision of about 99.7%, 99.7% recall, and F1 scores of approximately 97% across the 70 defect and non-defect test samples.

To continue demonstrating the robustness of the proposed method in learning and classifying the various kinds of defect and non-defect data samples in manufacturing products, another casting dataset was employed to train and test the introduced model. The results obtained from the experiments involving this dataset are shown in Table 4. According to the table, the presented method produced enhanced results against the other models, with Kp scores of 9.98 × 10^−1^, 1.00 × 10^+00^ MCC, and 1.00 × 10^+00^ accuracy. It also returned the least MSE of 6.70 × 10^−6^ and MSLE of 7.80 × 10^−8^, respectively.

**Figure 4:**
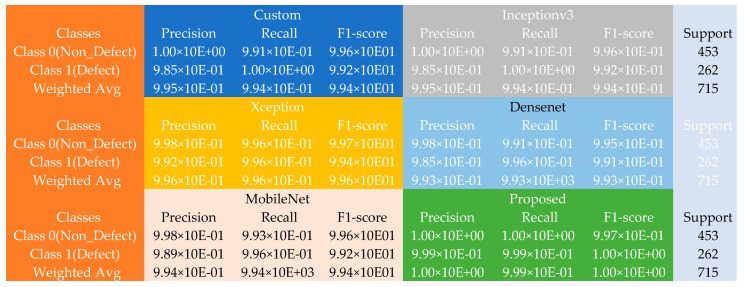
The confusion matrix table using the casting dataset.

Furthermore, the performance of the various models using the casting dataset is further showcased using the matrix table in Figure 4. Given the 453 non-defect and 262 defect samples, the proposed model returned an incredible performance improvement against the various pre-trained models and the custom model constructed during the experiments. The custom model produced weighted average precision, recall, and F1 scores of approximately 9.95 × 10^−1^ each across the 714 faulty and non-faulty test samples, with the Inceptionv3 model returning weighted average precision, recall, and F1 scores closely similar to the typical model. The Xception model, on the other hand, produced weighted average precision, recall, and F1 scores of about 9.96 × 10^−1^ each. At the same time, both the DenseNet returned weighted average precision, recall, and F1 scores of approximately 9.93 × 10^−1^ each across the 714 defect and non-defect test samples. However, the MobileNet returned weighted average precision, recall, and F1 scores of approximately 9.95 × 10^−1^ each across the 714 faulty and non-faulty test samples, and finally, the introduced approach displayed improved performance by returning better weighted average precision and F1 scores of about 1.00 × 10^+00^ and recall of approximately 9.99 × 10^−1^ across the 714 defect and non-defect test samples.

The PCB dataset was the final dataset used to train, test, and validate the proposed approach. As shown in Table 5, the proposed approach returned the enhanced results against the other models, with a Kp score of 9.78 × 10^−1^, 9.78^−1^ MCC, and 9.89 × 10^−1^ accuracy. The introduced method also outputted the lowest possible MSE score of 1.11 × 10^−2^ and MSLE of 5.34 × 10^−3^.

**Figure 5:**
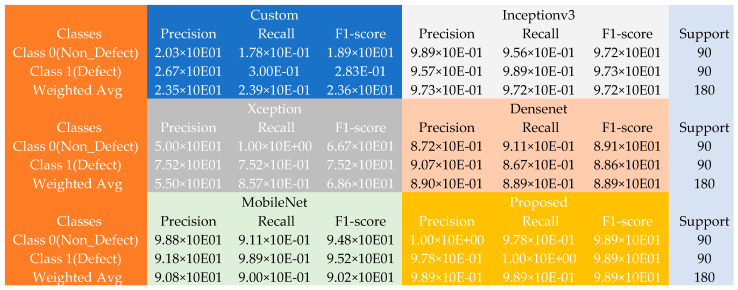
The confusion matrix table using the PCB dataset.

Finally, the performance of the different models using the PCB dataset was further demonstrated using the matrix table in Figure 5. With the 90 non-defect and 90 defect samples, the introduced model produced a remarkable performance improvement against the other pre-trained models and the unique conventional model constructed during the investigations. The custom model generated the lowest weighted average precision, recall, and F1 scores of approximately 2.35 × 10^−1^, 2.39 × 10^−1^, and 2.36 × 10^−1^, respectively, across the 180 faulty and non-faulty PCB test samples. The Inceptionv3 model returned weighted average precision, recall, and F1 scores of about 9.73 × 10^−1^, 9.72 × 10^−1^, and 9.72 × 10^−1^, respectively, while the Xception model produced weighted average precision, recall, and F1 scores of approximately 5.50 × 10^−1^, 8.57 × 10^−1^, and 6.86 × 10^−1^ respectively. Additionally, the DenseNet returned weighted average precision, recall, and F1 scores of approximately 8.90 × 10^−1^ each across the 180 defect and non-defect PCB test samples. In contrast, the MobileNet returned weighted average precision of 9.08 × 10^−1^, 9.00 × 10^−1^ recall, and 9.02 × 10^−1^ F1 scores. Finally, the proposed approach demonstrated an improved performance by producing superior weighted average precision, recall, and F1 scores of about 9.89 × 10^−1^ each across the 180 defect and non-defect PCB test samples.

**Figure 6:**
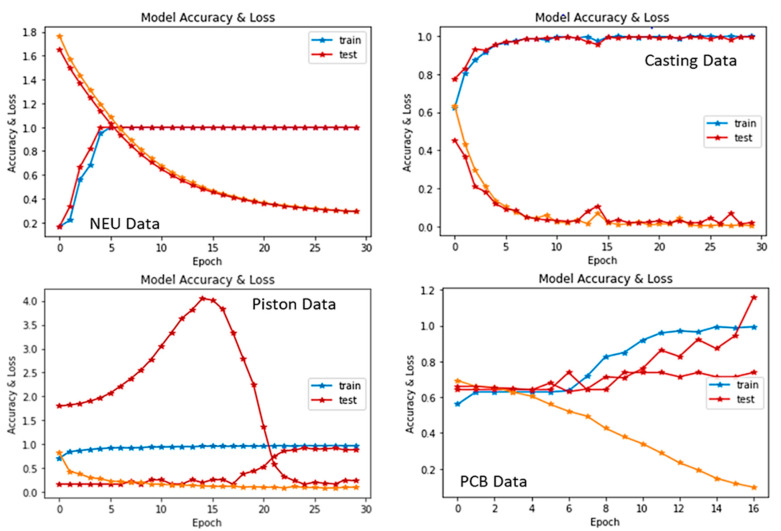
The output summary of the model training and testing process with the datasets.

We also compared our results with other similar studies in the literature. Our model outperformed ShuffleDefectNet [38], which used the ShuffleNet to detect metallic surface defects on the Northeastern University (NEU) dataset. While their method achieved a mean average accuracy of 99.75%, our proposed method returned an approximately mean weighted average of 100%. The introduced method in this work also outclassed the approach that combined a modified AlexNet architecture and support vector machine algorithm to classify the steel strip defect NEU dataset that yielded 99.7% accuracy [39]. For the classification of faulty and non-faulty samples of the PCB dataset, our method also outperformed the 98.79% accuracy score recorded by Adibhatla et al. [40], 97.5% by Khalilian et al. [41], 98% score by Kim et al. [42], and 98.1% by Bhattacharya and Cloutier [43] (see Figure 6).

Additionally, our introduced method performed better than the 98.06% accuracy score obtained by [44,45] and the 99% accuracy score recorded by Tang et al. [46] in the defect and non-defect piston data classification. Our result also surpassed the 95.5% accuracy score obtained by Lin et al. [47], with a 99.9% accuracy, 9.98 × 10^−1^ Kp score, and 1.00 × 10^+00^ MCC score, respectively, for the identification and separation of the defect and non-defect submersible pump impeller casting data samples. Hence, it can be inferred that our proposed method can achieve high accuracy in industrial implementation with high robustness in different defect and non-defect types.

## 6. Conclusions

This paper introduced an ensembled deep learning model for accurately and rapidly identifying and classifying defects and non-defects from manufactured industrial products. During the experiments in the study, different DL models were trained to individually learn the vital features necessary for distinguishing the faulty industrial products from non-faulty ones and then unifying them using the convolution LSTM sequence-based ensemler to obtain high accuracy and near-optimal models for inferencing and implementation. Different matrices were employed to test and validate the proposed model, with remarkable results obtained to support the usefulness of the new method.

The proposed model yielded a superior performance because of the fine-tuning process of the different parameters in the different adopted models used in the experiment with the specific datasets and the fusion of the features from the various participating models to overcome the drawbacks of the individual models. The results from the multiple experiments showed that the adopted DL networks yielded good generalization on the NEU dataset, and they are highly adaptable for transfer into different domains. The same characteristics exhibited on the NEU dataset were also repeated on the piston dataset by the various adopted models. However, the Xception and the proposed model returned better performance than the custom, Inceptionv3, DenseNet, and MobileNet on the casting dataset. On the other hand, the Inceptionv3, MobileNet, and the introduced model performed better than the custom, DenseNet, and Xception DL networks on the PCB data samples.

The ensembled architectures offered the proposed model great assistance in aggregating the fine features from the different classes of the datasets used in the experiments and, in turn, returned better results. The proposed model extracted the defect and non-defect features from the data samples pertinent to learning to distinguish faulty and non-faulty manufactured products. Our introduced method offers a general ensembling architecture for learning more deep feature representations from different and diverse datasets for robustness and better performance.

## Figures and Tables

**Table 1:** The conventional base model.

Layer Type	Output Shape	Parameters
conv2d (Conv2D)	(None, 45, 45, 32)	320
max_pooling2d	(None, 22, 22, 32)	0
conv2d_1 (Conv2D)	(None, 11, 11, 64)	18,496
max_pooling2d_1	(None, 5, 5, 64)	0
flatten (Flatten)	(None, 1600)	0
dense (Dense)	(None, 128)	204,928
dense_1 (Dense)	(None, 1)	129

**Table 2:** The classification report for each of the models and the final model using the NEU dataset.

	Kp	MCC	Accuracy	MSE	MSLE
Inceptionv3	9.93 × 10^−1^	9.93 × 10^−1^	9.94 × 10^−1^	4.72 × 10^−2^	3.58 × 10^−3^
Custom	9.99 × 10^−1^	9.95 × 10^−1^	9.94 × 10^−1^	4.21 × 10^−2^	3.49 × 10^−3^
DenseNet	9.99 × 10^−1^	9.94 × 10^−1^	9.94 × 10^−1^	4.53 × 10^−2^	2.78 × 10^−3^
MobileNet	9.94 × 10^−1^	9.98 × 10^−1^	9.93 × 10^−1^	3.17 × 10^−2^	2.34 × 10^−3^
Proposed	9.99 × 10^−1^	1.00 × 10^+00^	1.00 × 10^+00^	3.47 × 10^−4^	3.48 × 10^−6^

**Table 3:** The classification report for each of the models and the final model using the piston dataset.

	Kp	MCC	Accuracy	MSE	MSLE
Custom	8.51 × 10^−1^	8.61 × 10^−1^	9.43 × 10^−1^	5.71 × 10^−2^	2.75 × 10^−2^
Inceptionv3	7.90 × 10^−1^	7.90 × 10^−1^	9.14 × 10^−1^	8.57 × 10^−2^	4.12 × 10^−2^
Xception	9.66 × 10^−1^	9.66 × 10^−1^	9.86 × 10^−1^	1.43 × 10^−2^	6.87 × 10^−3^
Densenet	7.46 × 10^−1^	7.79 × 10^−1^	9.10 × 10^−1^	1.00 × 10^−1^	4.61 × 10^−2^
MobileNet	7.66 × 10^−1^	7.69 × 10^−1^	9.00 × 10^−1^	1.00 × 10^−1^	4.81 × 10^−2^
Proposed	9.80 × 10^−1^	9.72 × 10^−1^	9.49 × 10^−1^	2.89 × 10^−2^	2.93 × 10^−2^

**Table 4:** The classification report for each of the models and the final model using the casting dataset.

	Kp	MCC	Accuracy	MSE	MSLE
Custom	9.88 × 10^−1^	9.88 × 10^−1^	9.94 × 10^−1^	5.59 × 10^−3^	2.69 × 10^−3^
Inceptionv3	9.88 × 10^−1^	9.88 × 10^−1^	9.94 × 10^−1^	5.59 × 10^−3^	2.69 × 10^−3^
Xception	9.91 × 10^−1^	9.91 × 10^−1^	9.96 × 10^−1^	4.20 × 10^−3^	2.02 × 10^−3^
DenseNet	9.85 × 10^−1^	9.94 × 10^−1^	9.93 × 10^−1^	4.53 × 10^−2^	2.78 × 10^−3^
MobileNet	9.88 × 10^−1^	9.88 × 10^−1^	9.94 × 10^−1^	5.59 × 10^−3^	2.69 × 10^−3^
Proposed	9.98 × 10^−1^	1.00 × 10^+00^	1.00 × 10^+00^	6.70 × 10^−6^	7.80 × 10^−8^

**Table 5:** The classification report for each of the models and the final model using the PCB dataset.

	Kp	MCC	Accuracy	MSE	MSLE
Custom	8.52 × 10^−1^	7.83 × 10^−1^	8.39 × 10^−1^	2.76 × 10^−1^	1.66 × 10^−1^
Inceptionv3	9.44 × 10^−1^	9.45 × 10^−1^	9.72 × 10^−1^	2.78 × 10^−2^	1.34 × 10^−2^
Xception	7.97 × 10^−1^	8.11 × 10^−1^	8.96 × 10^−1^	4.72 × 10^−1^	2.40 × 10^−1^
DenseNet	7.78 × 10^−1^	7.79 × 10^−1^	8.89 × 10^−1^	1.11 × 10^−1^	5.34 × 10^−2^
MobileNet	9.00 × 10^−1^	9.03 × 10^−1^	9.50 × 10^−1^	5.00 × 10^−2^	2.40 × 10^−2^
Proposed	9.78 × 10^−1^	9.78 × 10^−1^	9.89 × 10^−1^	1.11 × 10^−2^	5.34 × 10^−3^

## Data Availability

(1) NEU (Northeastern University) surface defect dataset [34]. (2) PCB defect dataset [36]. (3) https://www.kaggle.com/datasets/ravirajsinh45/real-life-industrial-dataset-of-casting-product, accessed on 12 August 2022. (4) https://www.kaggle.com/datasets/satishpaladi11/mechanic-component-images-normal-defected, accessed on 12 August 2022.
